# Crohn’s disease presenting with pleural effusion: a case report

**DOI:** 10.1093/omcr/omae113

**Published:** 2024-10-10

**Authors:** Harem K Ahmed, Dilan S Hiwa, Soran H Tahir, Rawa M Ali, Dana T Gharib, Hoshmand R Asaad, Karokh F Hamahussein, Ayoob A Mohammed, Kayhan A Najar, Fahmi H Kakamad

**Affiliations:** Scientific Affairs Department, Smart Health Tower, Madam Mitterrand Street, Sulaymaniyah 46001, Kurdistan, Iraq; Shar Teaching Hospital, Internal Medicine Department, Malik Mahmud Ring Road, Sulaymaniyah 46001, Kurdistan, Iraq; Scientific Affairs Department, Smart Health Tower, Madam Mitterrand Street, Sulaymaniyah 46001, Kurdistan, Iraq; Scientific Affairs Department, Smart Health Tower, Madam Mitterrand Street, Sulaymaniyah 46001, Kurdistan, Iraq; College of Medicine, University of Sulaimani, Madam Mitterrand Street, Sulaymaniyah 46001, Kurdistan, Iraq; Scientific Affairs Department, Smart Health Tower, Madam Mitterrand Street, Sulaymaniyah 46001, Kurdistan, Iraq; Department of pathology, Hospital for Treatment of Victims of Chemical Weapons, Hamawghan road, Halabja 46006, Kurdistan, Iraq; Scientific Affairs Department, Smart Health Tower, Madam Mitterrand Street, Sulaymaniyah 46001, Kurdistan, Iraq; Gastroenterology Department, Gastroenterology and Hepatology Teaching Hospital, Zanko street, Sulaymaniyah 46001, Kurdistan, Iraq; Scientific Affairs Department, Smart Health Tower, Madam Mitterrand Street, Sulaymaniyah 46001, Kurdistan, Iraq; Gastroenterology Department, Gastroenterology and Hepatology Teaching Hospital, Zanko street, Sulaymaniyah 46001, Kurdistan, Iraq; Scientific Affairs Department, Smart Health Tower, Madam Mitterrand Street, Sulaymaniyah 46001, Kurdistan, Iraq; Gastroenterology Department, Gastroenterology and Hepatology Teaching Hospital, Zanko street, Sulaymaniyah 46001, Kurdistan, Iraq; Scientific Affairs Department, Smart Health Tower, Madam Mitterrand Street, Sulaymaniyah 46001, Kurdistan, Iraq; College of Medicine, University of Sulaimani, Madam Mitterrand Street, Sulaymaniyah 46001, Kurdistan, Iraq; Scientific Affairs Department, Smart Health Tower, Madam Mitterrand Street, Sulaymaniyah 46001, Kurdistan, Iraq; Research department, Kscien Organization for Scientific Research, Hamdi Street, Azadi Mall, Sulaymaniyah 46001, Kurdistan, Iraq; Scientific Affairs Department, Smart Health Tower, Madam Mitterrand Street, Sulaymaniyah 46001, Kurdistan, Iraq; College of Medicine, University of Sulaimani, Madam Mitterrand Street, Sulaymaniyah 46001, Kurdistan, Iraq; Research department, Kscien Organization for Scientific Research, Hamdi Street, Azadi Mall, Sulaymaniyah 46001, Kurdistan, Iraq

**Keywords:** Crohn’s disease, inflammatory bowel disease, pulmonary, pleural effusion, case report

## Abstract

Crohn’s disease (CD) is a granulomatous inflammatory bowel disease. Around 25% of CD patients exhibit extraintestinal manifestations, though pulmonary involvement is rare. This study presents a case of CD causing pleural effusion. A 43-year-old man visited the pulmonology clinic with a dry cough for one-month, right-side pleuritic chest pain, and exertional dyspnea. He was treated with antihistamines and antitussive syrup, with incomplete relief. A chest CT scan showed bilateral mild pleural effusion. Given his occasional black stools and high serum calprotectin, a colonoscopy confirmed CD. Pulmonary manifestations may involve airway, parenchymal, and interstitial pathologies, but no distinctive pathological findings differentiate CD’s pulmonary manifestations from other causes. In conclusion, isolated bilateral pleural effusion and the underlying pleuritis as a pulmonary manifestation of CD—characterized by dry cough and pleuritic chest pain, particularly preceding the diagnosis of CD—is extremely rare but possible.

## Introduction

Crohn’s disease (CD) constitutes a granulomatous form of inflammatory bowel disease, manifesting with inflammation and possessing the capacity to affect any segment within the gastrointestinal tract, in particular, the terminal ileum and colon emerge as the predominantly involved regions. The hallmark features encompass a segmental and discontinuous inflammatory reaction involving all the layers of the intestinal wall. The etiology of CD is postulated to stem from the complex interplay of environmental factors, intestinal microflora, and genetic susceptibility. This multifaceted interaction precipitates an aberrant mucosal immune response and compromises the integrity of the epithelial barrier [1, 2].

Nearly a quarter of patients with CD can be observed to exhibit extraintestinal manifestations including, but not limited to, erythema nodosum, anterior uveitis, episcleritis, peripheral and axial arthropathy, primary sclerosing cholangitis, and pyoderma gangrenosum [3]. Pulmonary manifestations are rarely encountered in individuals with CD, encompassing a spectrum that extends from asymptomatic and latent conditions to critical medical presentations. The underlying mechanism of respiratory implications in CD remains incompletely explained. A plausible association is proposed by development through a mutual embryologic derivative from the primordial foregut, a shared developmental origin for both the pulmonary and gastrointestinal systems. In the review of genuine literature, in the majority of cases of pulmonary involvement, the pulmonary symptoms manifest after the diagnosis of CD [4–7]. We herein report a case of CD that presented with pulmonary manifestation in the form of bilateral pleural effusion that preceded the diagnosis of CD by a few weeks.

## Case presentation

### Clinical presentation

A 43-year-old male agricultural engineer presented to the pulmonology clinic complaining of a dry cough for one month associated with right-sided pleuritic chest pain, exertional dyspnea, muscle pain, and easy fatigability. He also noticed a significant weight loss of approximately 9 kg since the beginning of his symptoms. On further questioning, he mentioned occasional black stools for the last few months associated with constipation. The patient had no history of contact to patient with active pulmonary tuberculosis, but he had a personal history of migraine and a family history of colorectal carcinoma.

### Diagnostic approach

Physical examination revealed no significant finding however, initial investigations revealed normochromic microcytic anemia (hemoglobin 12.1 g/dl). There was an elevated erythrocyte sedimentation rate (ESR) of 42 mm/h (after correction to his mild anemia). Liver enzymes were also mildly elevated, alanine aminotransferase (ALT) level of 69.6 IU/l.

**Figure 1:**
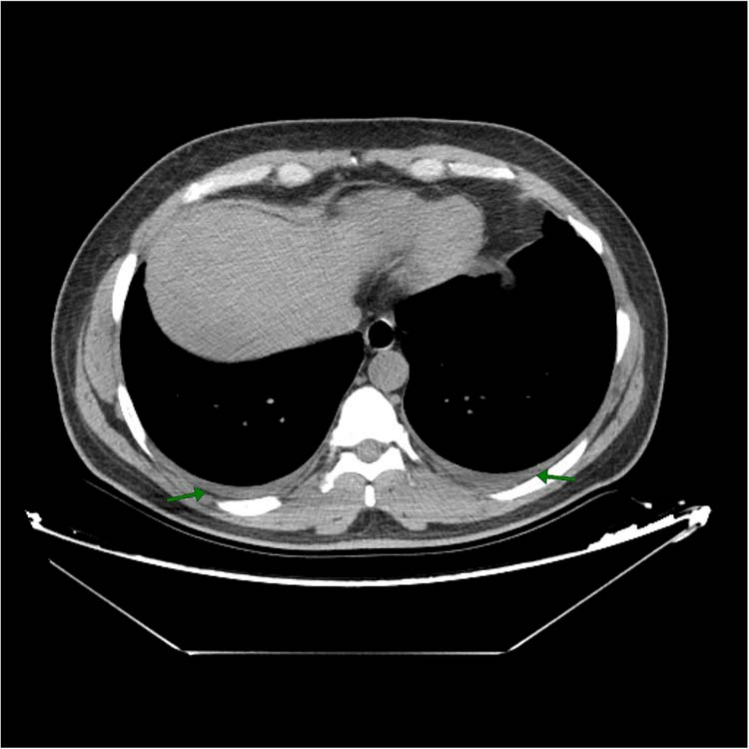
Axial section of native chest CT scan shows bilateral mild pleural effusion with arrows indicating the affected areas.

**Figure 2:**
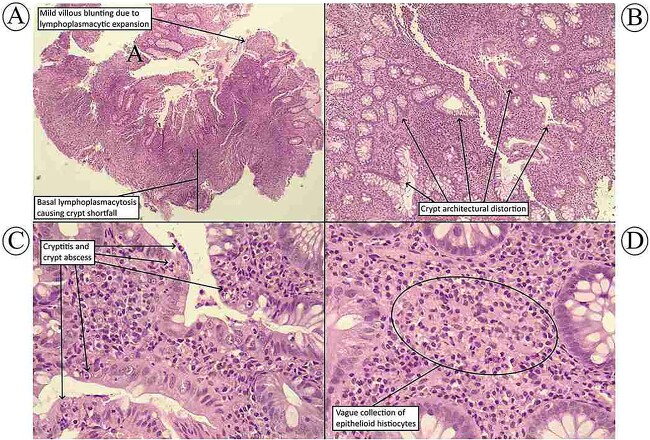
(**A**) The terminal ileal biopsy shows mild villous blunting due to lymphoplasmacytic expansion of the lamina propria. The inflammation extends deeply causing a prominent elevation of the crypt bases (crypt shortfall). (**B**) The colonic biopsy shows glandular architectural distortion in the form of uneven size, shape, and distance between the crypts. (**C**) There are prominent neutrophilic cryptitis and crypt abscesses within the colonic biopsy. (**D**) There is a vague collection of epithelioid histiocytes in the lamina propria of the colon. [Hematoxylin and eosin stain; magnification of 40× (**A**), 100× (**B**), and 400× (C and D)].

The patient received a course of antihistamines and an antitussive syrup. However, these demonstrated an incomplete response in mitigating the symptoms, prompting imaging and spirometry to be performed for a more comprehensive assessment. A computed tomography (CT) scan of the chest revealed no Ghon’s complex, cavitation, or consolidation to suggest either primary or secondary activation of pulmonary tuberculosis, it only showed bilateral mild pleural effusion with subtle fibrotic bands in the left upper lobe (Fig. 1). It is worth mentioning that there was no other sign of embolism, like interstitial edema or consolidation. More importantly, the patient had no risk factor for pulmonary embolism and had an “unlikely” score of pulmonary embolism on modified Well’s score. The spirometry pulmonary function tests showed a forced vital capacity (FVC) of 83% predictive value and forced expiratory volume in 1 s (FEV1) of 86% predictive value. Echocardiography revealed no abnormality. Renal function tests were within the normal range, and the connective tissue screen and viral hepatitis screen were all negative. Ferritin level was low (18.37 ng/ml), and the fecal calprotectin level was very high (435.46 μg/g). After consultation with a gastroenterologist, a decision was made to perform endoscopy. Esophago-gastro-duodenoscopy revealed gastric body mucosal thickening, pre-pyloric erosions, and a few bulbar erosions. Biopsies taken from the stomach according to Sydney protocol showed mild chronic inactive H.pylori gastritis. There were no endoscopic and histopathological features to suggest Crohn’s disease. However, colonoscopy revealed a patchy distribution of inflammation with skip lesions of moderate severity throughout the colon and terminal ileum, which were consistent with active CD. Histopathological examination of the biopsy showed chronic active ileo-colitis with vague, small collections of histiocytes, consistent with the diagnosis of CD (Fig. 2). There were no well-formed, necrotizing granulomas to raise suspicion of gastrointestinal tuberculosis.

### Therapeutic intervention and outcome

Azathioprine tablet (50 mg twice daily), prednisolone tablet (20 mg daily) with a tapering plan, antihistamine, and an oral proton pump inhibitor were prescribed. The patient experienced a dramatic response and significant improvement in terms of his pulmonary and gastrointestinal symptoms, accompanied by the normalization of his diagnostic investigation parameters.

## Discussion

Pulmonary involvement in CD can encompass a spectrum that extends from asymptomatic and latent conditions to critical medical presentations. The occurrence of subclinical lung involvement is notably higher than the manifestation of overt respiratory symptoms, documented in as much as 30% to 60% of individuals diagnosed with CD. Individuals with inflammatory bowel disease who do not exhibit respiratory symptoms have been documented to have abnormalities in pulmonary function tests in nearly 40% of the patients, in stark contrast to the 3% prevalence observed in the control group. Furthermore, in up to 50% of individuals with CD, bronchoalveolar lavage discloses the presence of alveolitis, even in the absence of evident clinical respiratory symptoms. Remarkably, the aforementioned alterations endure consistently even within the context of clinical remission [4, 8].

The most prevalent complaints that patients present with are dry cough, progressive shortness of breath, and chest pain, findings present in the current case. Flu-like symptoms and fever have been reported as well. The potential respiratory manifestations could be the result of airway involvement in the form of chronic bronchitis, bronchiectasis, chronic bronchiolitis, and tracheal obstruction. In addition, parenchymal and interstitial pathologies of the lungs have been reported, such as pulmonary eosinophilia, bronchiolitis obliterans organizing pneumonia, noncaseating granulomatous inflammation, and fibrosis. It is worth mentioning that only a limited number of cases detailing pleural manifestation in CD have been documented in the literature. Pleural involvement can be categorized into pleural thickening, pneumothorax, pleuritis, and pleural effusion. Isolated pleural effusion is an infrequent presentation, with a higher prevalence of pericarditis in these cases. It is postulated that the etiology of pericarditis lies in the deposition of circulating immune complexes, which emanate from the patient’s intestine and accumulate in the pericardium. This process is considered the causative factor in the onset of pericarditis. However, there was only bilateral pleural effusion with no associated pericarditis in the present case, adding to the rarity of this patient’s presentation. The pleuritic chest pain in the current case was probably due to the pleuritis of inflammatory origin that preceded the development of mild pleural effusion. The serositis underlying the effusion typically manifests as an exudative rather than transudative effusion, a distinction more commonly associated with extra-thoracic diseases. This differentiation can aid in refining the differential diagnosis for these patients [3, 9].

Currently, no distinctive pathological findings have been reported that set apart pulmonary extraintestinal manifestations of CD from other potential causes. It is important to note that drug-induced lung pathology must be kept in the differential diagnosis of patients with CD taking medications such as methotrexate, sulfasalazine, anti-tumor necrosis factor (TNF)-alpha, and mesalamine, as this was shown to be responsible for 50% of interstitial lung diseases in individuals diagnosed with inflammatory bowel disease [2].

The question of administering therapy to asymptomatic patients remains uncertain. Nonetheless, it is imperative to consider that individuals with inflammatory bowel disease have an elevated risk of mortality associated with pulmonary ailments. Efficacious and enduring responses are commonly observed in most patients with airway and parenchymal pathology following the administration of systemic or inhaled steroids. The adjunctive use of cyclophosphamide or infliximab has demonstrated the capacity to evoke a rapid and favorable clinical and radiological response in select cases. The administration of prednisone has exhibited significant clinical efficacy in targeting pleural involvement in CD. The present case demonstrated a favorable outcome with the administration of 20 mg prednisone tablets in addition to 50 mg azathioprine twice daily with the purpose of treating his active CD as well. Nonetheless, more research is required to determine whether prednisone alone is sufficient or whether the addition of azathioprine was helpful in the prompt resolution of the cough and pleuritic chest pain of the patient. Moreover, pleural drainage may be necessary if there is no clinical improvement. In some cases, invasive interventions, for instance, thoracoscopy and bronchoscopy, may be essential to attain a conclusive diagnosis [5, 10].

One of the limitations of this study is the lack of cytologic, biochemical, and immunologic analysis of the pleural effusion fluid that could have added more information to the literature regarding the underlying pathophysiology of this condition because it was not deemed clinically necessary for managing the patient.

In conclusion, isolated bilateral pleural effusion and the underlying pleuritis as a pulmonary manifestation of CD—characterized by dry cough and pleuritic chest pain, particularly preceding the diagnosis of CD—is extremely rare but possible. This condition requires a high degree of clinical suspicion. Treatment with prednisone and azathioprine may effectively control respiratory symptoms.

## Data Availability

Not applicable.
